# Feasibility and Acceptability of a Multimedia Childbirth Education Intervention for Black Women and Birthing People and Their Birth Companions

**DOI:** 10.3390/healthcare13101106

**Published:** 2025-05-09

**Authors:** Soroya Julian McFarlane, Tamora Callands, Diane B. Francis, Andrea Swartzendruber, Divya S

**Affiliations:** 1 Department of Communication Studies, University of Georgia (UGA), Athens, GA 30602, USA; divya.s@uga.edu; 2 College of Public Health, University of Georgia (UGA), Athens, GA 30602, USA; tamcall@uga.edu (T.C.); swartz@uga.edu (A.S.); 3 College of Arts, Media and Design, Northeastern University, Boston, MA 02115, USA; di.francis@northeastern.edu

**Keywords:** maternal health, health communication, health disparities, Black/African American

## Abstract

**Background/Objectives:** This study aimed to evaluate a novel, theory-informed multimedia learning intervention (an animation and a game) designed to target Black Women and Birthing People (BWBP) and their companions as catalysts for change in improving maternal health communication disparities. **Methods**: We used an explanatory sequential mixed-method design to examine the feasibility and acceptability of the intervention. A total of 135 BWBP completed a survey; 14 participated in follow-up interviews. **Results**: The participants reported high levels of feasibility, acceptability, and appropriateness of the intervention. The knowledge scores improved significantly after exposure to the animation and game; the intentions to use and share the intervention were also high. The participants scored moderately for agency and self-efficacy after exposure to the intervention, suggesting that there may still be a need for more messages to support BWBP. **Conclusions**: These promising results lay a foundation for elucidating the role of communication in addressing maternal health disparities and demonstrates the importance of a holistic approach to maternal health that intervenes at the community level (via the family care team) to impact the interaction with the institution level (the healthcare team).

## 1. Introduction

Despite alarming disparities in maternal health outcomes for Black women and birthing people (BWBP) compared to their White counterparts, institutional childbirth education is not tailored according to cultural needs and preferences [1]. Black women are 3–5 times more likely to suffer pregnancy-related mortality compared to White women [2], but White women are two times more likely to have attended birth classes offered by hospitals [3]. Childbirth education is positively associated with several outcomes, including maternal knowledge, reduced anxiety, increased confidence and satisfaction with birth experiences, informed decision-making, and a potentially decreased likelihood of medical interventions during labor [4]. BWBP without adequate and relevant information become additionally vulnerable to suffering related maternal complications or poor health outcomes [5]. Though it is well established that racism and systemic inequities influence healthcare provider interactions [6,7,8], access to adequate and relevant knowledge as a dimension of reproductive justice is underexplored in public health and maternal health interventions. Reproductive justice is a framework that is rooted in Black feminist scholarship and extends beyond the right to choose (or not choose) pregnancy to include the right to parent in supportive and safe environments [9]. This lens also shifts away from biomedical and physician-centered approaches toward patient-centered and community-engaged approaches [10]. Evidence of the extent of public awareness and knowledge of this Black maternal health communication disparity is limited. We define Black maternal health communication disparities as differences in the amount and appropriateness of information shared by healthcare providers, based on identity, that lead to unnecessary birth trauma (i.e., disempowerment, communication dissatisfaction, depression, and other pregnancy-related illnesses).

In lieu of participating in hospital-based childbirth classes, BWBP tend to seek and access social support from family members [11] and require their mothers and other extended family members’ presence during childbirth more than their White counterparts [12]. Social support from trained and untrained personal aides is associated with reductions in the need for analgesia and a Caesarean section and increases in spontaneous vaginal delivery without interventions like forceps or vacuum extraction [13]. Specifically, Black doulas (birth coaches) may be an important source of support; recent evidence suggests that their communication and advocacy skills may be transferable to BWBP’s chosen companions for labor and delivery [14]. While it is well established that childbirth education can influence birth outcomes, there is a dearth of education interventions targeting BWBP and the persons who provide social support for BWBP [15]. The purpose of the current study was to evaluate the feasibility, acceptability, and appropriateness of an education, communication, and advocacy intervention for birth companions as facilitators to reduce unnecessary BWBP birth trauma.

### 1.1. Background

The vast majority of expectant parents (98.4%) in the United States opt into standardized prenatal treatment and care within a hospital-based system [16]. Since pregnant people depend on their providers for information, most maternal healthcare systems include childbirth education as a part of routine prenatal care. These classes are offered in various formats and modalities, and usually include information on the fetal development in utero, the process of labor, pain management options and techniques, options for clinical interventions, what to expect and in terms of hospital care during labor and delivery, and postpartum advice and offerings (for example, lactation consultants and support groups) [4,17,18]. The effectiveness of these programs can vary based on individual needs and the level of engagement from expectant parents [14]. Only 18% of pregnant people participate in these institutional childbirth classes [1]. The COVID-19 pandemic exacerbated the problem of low engagement with traditional childbirth education; even with improvements in online offerings for expectant parents, only about half of those who participated chose this option [19].

“Culture is among the most significant variables that influence a woman’s perception of the childbearing experience” [20]. Institutional childbirth education is not considered a priority for BWBP, who believe that they have innate knowledge and skills for childbirth, or can access this through female relatives and important others in their community [18]. BWBP also feel stigmatized if they attend in-person birth classes because of their marital status [18]. There is ample evidence to support the fact of medical providers’ stereotyping of Black pregnant patients based on their educational attainment and marital and insurance statuses [21,22]. Childbirth education classes are also less accessible to BWBP due to reasons such as their insurance status, a lack of childcare, and unreliable transportation [23].

Community-based doulas are associated with providing continuity in care, acknowledging identity and combating systemic racism, increasing agency and reductions in the need for clinical interventions [24,25,26]. Doulas’ care also leads to higher self-efficacy and maternal satisfaction and wellbeing [13,27,28]. However, doulas are not covered by insurance in many states [27,29]. As such, doulas are not accessible to most BWBP; only 6% of birthing people have doulas [26], with the vast majority of doula clients being White women [30,31].

With questions about the continued appropriateness of hospital-based classes [1], online programs may be a method for improving accessibility and relevance. They provide flexibility in timing and location, enabling expectant parents to access information at their convenience [1]. They also allow for cultural tailoring of messages. Integrating elements of online and community-based approaches might offer a comprehensive and culturally appropriate childbirth education experience for BWBP [32].

### 1.2. The Intervention and Current Study: Animation and Game

**Figure 1:**
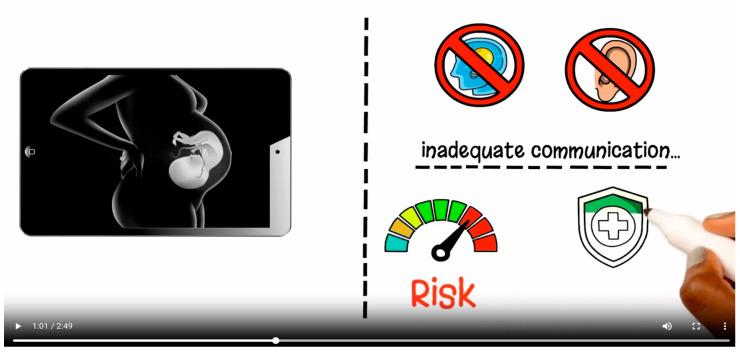
Screenshot of the animation.

**Figure 2:**
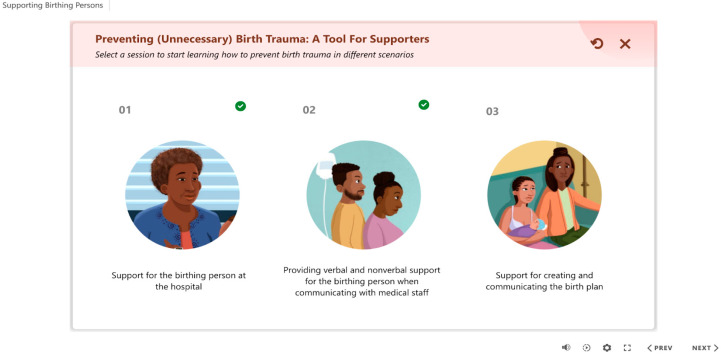
Screenshot of scenarios in the game.

A team of multidisciplinary researchers designed multimedia assets to address a gap in the communication interventions targeting birth companions. The assets, in the form of an animation and game (i.e., interactive storyboard), were based on extant literature on Black maternal health, as well as the authors’ formative research (interviews and focus groups with Black birthing people and Black doulas) [14]. The intervention was co-designed via consultations with a Black doula community-based organization and a clinical/research expert in obstetrics/gynecology with expertise in Black maternal health disparities. The intervention was also designed based on constructs from communication and psychological theories, including the Agency-Identity model. The intervention includes messages that educate birthing people and their companions about disparities in health outcomes for Black women and birthing people and aims to promote social support. The game uses scenarios to allow viewers to role-play and learn specific social support skills that would be important while navigating the delivery hospital. The animation and game were available on a mobile friendly website (http://thethriveproject.org, accessed on 6 May 2025) (see Figure 1 and Figure 2). In this paper, we report the findings from a preliminary testing of the multimedia intervention.

## 2. Materials and Methods

We used an explanatory sequential mixed-method design to examine the feasibility and acceptability of the multimedia intervention. Quantitative data collection and analyses informed the qualitative data collection and analysis. Using both quantitative (i.e., survey) and qualitative (i.e., interview) methods enabled the study team to obtain a more comprehensive account of the perceptions of the intervention. The University of Georgia granted ethical approval for this study.

### 2.1. Participants and Procedures

Our sample was recruited through an online panel maintained by the research organization Qualtrics. We employed a non-probability purposive sampling approach to recruit potential participants. They were eligible for the study if they were 18–44 years old, resided in the United States of America, identified as Black women, and had a live birth between 2013 and 2023. Qualtrics Panel is a managed research service that draws from a large pool of pre-screened panelists who voluntarily opt in to complete surveys. Qualtrics uses demographic targeting based on the researcher’s criteria and distributes the survey link to eligible panelists. The service also ensures quality control by preventing duplicate responses and screening for inattentive or fraudulent responses. The target sample size (n = 135) was selected based on feasibility and our goal of capturing diverse perspectives across demographic groups. After giving informed consent, Qualtrics provided the participants with a link to the survey study. The multimedia intervention components (i.e., animation and game) were embedded within the online survey questionnaire. The participants first completed a set of questions intended to gauge their knowledge about Black women and birthing people’s pregnancy and childbirth experiences. They then saw and responded to questions about the animation, followed by playing the game and responding to a set of questions. The knowledge questions were repeated after exposure to the intervention materials. After completing the survey, the participants were asked if they would be willing to participate in a semi-structured interview with the study team. Willing participants were asked to provide their contact information. This information was used to compile a list of potential participants. Two trained research assistants reached out to the potential interviewees to set up dates and times for the interview. The interviews were held on Zoom and lasted up to 60 min.

A total of 135 Black women and birthing people completed the survey; 14 participated in the follow-up interviews. The survey participants ranged in age from 18 to 44 years; 48% were aged 24–34 years. The mean age was 29.79 (SD = 6.76). Table 1 provides participant demographics information. Seventy percent of the participants had some college education or degree and 51% reported a household income of less than USD 45,000. Most were either unmarried with a partner (53%) or married (37%). The participants had an average of 2.1 children (range = 1–5). A third of the participants were from three states (Texas, Louisiana, and Georgia). The sample’s demographic and geographic composition aligns with the national patterns observed among BWBP most affected by these disparities—particularly those living in Southern states such as Georgia, Louisiana, and Texas. These states have historically high Black maternal mortality rates and serve as priority regions for intervention and research. The participants’ educational and income characteristics also reflect the diversity within the broader BWBP population that seeks community-based or publicly funded maternal care (https://www.marchofdimes.org/sites/default/files/2024-11/US_Report_Card_2024_English.pdf, accessed on 6 May 2025).

### 2.2. Survey Measures

The study adapted a prior scale to assess the feasibility, acceptability, and appropriateness of the intervention [30]. Each was measured with four items on a 5-point Likert scale (1 = strongly disagree to 5 = strongly agree). For feasibility, the participants answered whether the intervention met their approval and appealed to them and if they liked and welcomed the intervention. Acceptability measured whether the intervention seemed fitting, suitable, applicable, and like a good match. Intervention appropriateness measured whether the intervention seemed implementable, possible, doable, and easy to use.

Knowledge was assessed initially using 7 questions developed by the researchers based on information that was included in the animation and the game. The participants were asked to mark if each statement was true, false, or they were unsure. Correct responses were coded as 1, incorrect coded as 0, and all responses were summed to create a scale such that higher scores indicated that more questions were answered correctly. However, after examination of the individual items, the 3 recoded items were removed because the researchers deemed them problematic. These recoded items were the only items that participants’ knowledge scores decreased, which supports recent literature suggesting that recoded items may be unreliable and should no longer be included surveys [31,32,33].

We also assessed agency, self-efficacy, and intentions. Agency was measured with seven items (e.g., “I am comfortable voicing my views, opinions and beliefs to my maternal healthcare providers” and “I am comfortable with, and proud of, who I am when interacting with my maternal healthcare providers”). The responses were given on a seven-point scale (1 = strongly disagree to 7 = strongly agree). Self-efficacy was measured with three items, for example, “I am aware of, and confident in, my strengths and abilities when interacting with my maternal healthcare providers”. The responses were on a seven-point scale (1 = strongly disagree to 7 = strongly agree). Intentions to share were measured with five items; the participants were asked about their likelihood of discussing the animation or game with a birth companion, family member, friend, or healthcare provider, recommending it to someone they know, or sharing it online or on social media. The responses were given on a five-point scale (1 = extremely unlikely to 5 = extremely likely). Intentions to use were measured with a single item: “If you were to get pregnant in the future, how likely are you to use the animation or game tools to support your birth?” on a five-point scale (1 = extremely unlikely to 5 = extremely likely).

### 2.3. Interview Guide

Interview questions were developed based on the quantitative data which focused on the feasibility, acceptability, and appropriateness of the multimedia intervention. The majority of the interview questions were open-ended and probing questions were used as needed to clarify respondents’ responses. Example questions included the following: Regarding the animation, what do you think? What are your initial reactions? What did you like or enjoy about the video, and why? After playing the game, in what ways would you be able to rely on your birth companion for support during labor and delivery?

### 2.4. Data Analysis

Quantitative data from the questionnaires were analyzed and presented descriptively with summary statistics (means or percentages). Paired sample t tests were used to explore the changes in knowledge scores before and after exposure to the intervention. All analyses were conducted using SPSS statistical software (version 29).

Qualitative data were analyzed using rapid qualitative analyses that focused on data reduction activities. The interviews were recorded and transcribed. One of investigators reviewed all the transcripts and guided a team of five research assistants through the coding process. The investigator used thematic analyses to categorize the responses across all the interviews. Each interview was independently coded by two research assistants and then discussed in the larger team to ensure analytic rigor and validity. The analyses were conducted in Microsoft Word.

## 3. Results

The results reported below represent an integrated picture (quantitative and qualitative) of the feedback from the participants in our study. As such, we report survey and interview data alongside one another.

### 3.1. Feasibility, Acceptability, and Appropriateness

Overall, the multimedia intervention was well received. The participants reported high levels of feasibility of the animation (M = 4.3 SD = 0.79) and the game (M = 4.25 SD = 0.73). The ratings were similarly high for acceptability (animation M = 4.1 SD = 0.82; game M = 4.3 SD = 0.70) and appropriateness (animation M = 4.36 SD = 0.66; game M = 4.29 SD = 0.76). In the interviews, the participants reported that they liked that the multimedia intervention emphasized having a birth plan and advocated for sticking to the plan. The participants remarked that they appreciated that the material was not overly “scientific”, “easy to grasp”, and provided specific actions and questions that one can ask the person who is delivering the baby. Additionally, the participants indicated that the animation would be helpful for birthing companions. All the participants except one reported that they did not dislike anything about the game. When asked what participants did not like about the animation, most participants indicated that there was nothing they did not like. However, two participants did provide feedback for improvement. One participant reported that they wished the game expanded with different scenarios to include “low-income areas” and those on “Medicare programs”. Another one indicated that it would have been nice to have real-life scenarios from people’s lived experiences, and the other participant reported that the term “Black birthing people” had a “positive tone… and covered the goal… but it takes away from our womanhood”. This participant remarked that she hopes people will investigate it further before using it.

### 3.2. Knowledge

The knowledge scores improved significantly (<0.001) from before exposure to the animation and game play (M = 3.09, SD = 1.03) compared to after (M = 3.52, SD = 0.91). All the participants in the interviews reported that the animation was informative about Black women’s birthing outcomes and very relatable. Several women remarked that they were surprised or did not know that “3 in 5 Black women experience trauma and/or die during pregnancy”. There was a sentiment throughout the interviews that the information provided “shed light” on the poor outcomes linked to birthing for Black women that have been overlooked for years.

*One participant noted, “what caught my attention the most? Honestly, probably the part where it was saying how Black pregnant people are more likely to experience trauma”.*

(Participant 5, Line 217–18)

Furthermore, the participants remarked that the scenarios presented were relatable and brought attention to issues that the public may not know about which are impacting the Black birthing community such as importance of communication with hospital staff and doctors, and the rates of maternal mortality and trauma. In general, the participants shared that they appreciated the real-life scenarios presented in the game. The participants did not know that “fathers”, “daddies”, and support people can advocate for them as BWBP. One person remarked that next time, they will get their partner to take a doula class or become a doula—“they did not know men could be doulas”. Furthermore, they learned the benefits of positive words, a calming spirit, and breathing techniques and that support people can advocate for the BWBP (see Table 1, Table 2 and Table 3).

The participants felt that the best time during pregnancy to learn the information presented in the animation would be early (63% preferred early pregnancy, 30% preferred mid-pregnancy (28 weeks), and 7% preferred late pregnancy). For the game, the best time to learn the information was early (70% preferred early pregnancy, 21% preferred mid-pregnancy (28 weeks), and 8% preferred late pregnancy.

### 3.3. Agency

The participants had moderate scores for agency after exposure to the intervention (M = 5.22 SD = 1.14). However, when asked if they felt empowered after playing the game, all the participants remarked that they did feel more empowered. Specifically, the participants reported gaining or reinforcing awareness that they have ‘choices’, that ‘it is important to speak up for yourself’, and a clearer understanding of ‘the importance and role of the doula’.

### 3.4. Self-Efficacy

The participants had moderate scores for self-efficacy after exposure to the intervention (M = 5.75 SD = 0.57). Several participants remarked that the scenarios provided “in the moment” ways that one can respond to hospital staff. They indicated that they now know “you have options” during the labor and delivery process. In addition, several participants reported that they feel more confident in their ability to navigate the medication system and how to involve their partner as an advocate on their behalf with the medical team and as support for them as the birthing person during labor.

### 3.5. Intentions

The likelihood to share information was high (M = 3.46 SD = 0.57). Looking at the individual items in this scale, the participants were most likely to discuss the animation or game with their healthcare provider (M = 3.56, SD = 0.70), followed by discussing it with a birth companion (M = 3.51, SD = 0.69) and with family and friends (M = 3.47, SD = 0.69), recommend it to someone they know (M = 3.45, SD = 0.72), and share a link to it on social media (M = 3.32, SD = 0.87). The respondents unanimously agreed that the multimedia tool was effective and would highly recommend it to acquaintances and family members for its insights on the birthing process. They felt empowered and optimistic after using the multimedia tool and felt they would be better able to advocate for themselves if they had this resource. The respondents believed that sharing the resources and materials through an online medium would make it easily accessible to reach a wider audience.

The likelihood of using the animation or game for a future birth was high (M = 4.08 SD = 1.24). In the interviews, the respondents said

*“Yes, definitely. I definitely would [use this tool for a future birth or share it with others]. Just to let people have that information and that give them insight if there are other options. You don’t have to keep doing that routine that had been passed on from our moms, our grandmothers, and that same routine. There’re other options now that could help us”.*

(Participant 2)

*“…. I have little sisters and I think it’s something that if I was around a person, if I didn’t show them the game, I would at least share what I learned from it to try to help them through the process. I think it’s valuable information and people don’t talk about it. Now it’s starting to become a conversation”.*

(Participant 12)

## 4. Discussion

The results of the current study suggest that these multimedia assets are acceptable and feasible for providing an interactive web-based option to teach the communication and advocacy skills needed for Black birthing in hospitals in America. There are limited theory-driven and quantitative studies on maternal health (Dahlem et al., 2015 [34]). Both qualitative and quantitative findings have shown that BWBP continue to lack knowledge about the health disparities affecting them [29]. Here, the quantitative findings showed significant differences between the pre- and post-test scores on knowledge, especially the item that focused on the stark disparities in maternal mortality between White and Black birthing people. The interviews with a subset of the BWBP from the study provided further context on this item as it garnered attention and surprise. This lack of knowledge puts the community at even greater risk of poor outcomes as they are unprepared to navigate the current healthcare system (i.e., do not have the necessary skills). This suggests that it is important to share this message in campaigns aiming to motivate BWBP to engage more meaningfully and actively in their maternal care.

The results for self-efficacy and agency were only moderate after exposure to the intervention. This may be demonstrating that there is a need for additional messages and interventions that would increase self-efficacy and agency, which is in line with prior research [35]. In addition, the researchers used the term “Black women and birthing people” in the animation and game to be inclusive and in line with current recommendations [36]. However, some participants felt that the term “birthing people” was taking away from the “woman” identity. This feedback highlights the complexities of language use in health communication and underscores how terms intended to be inclusive can still be perceived as exclusionary or politically charged, depending on the context and audience. Rather than viewing this tension as a flaw, we see it as an opportunity to engage in ongoing ethical reflection. It is still unclear how to navigate this dialectic; we will continue to work with community partners to determine the best language to use and in what context (e.g., academic publications versus recruitment material) and continue to be guided by community leaders and experts around language use that is both inclusive and culturally responsive. Future iterations of the intervention will continue to reflect these evolving conversations.

The current study lays a foundation for elucidating the role of communication in addressing maternal health disparities. This multimedia approach can be used as a supplement and can serve as a practical tool for knowledge translation in various maternal health education contexts beyond hospital classes. They can be used by (1) doulas and midwives’ clients who operate independently, and (2) companions of Black birthing people who encompass a possible range of individuals such as family, friends, and partners who may also be racially diverse. The fact that these assets are online means that they can be easily shared by BWBP with their chosen companions and care team. While this study focused on the feasibility of the multimedia education intervention, the next step would be to determine the potential impact of exposure to the intervention on reducing unnecessary BWBP birth trauma.

We did not collect data on the participants’ prior childbirth education experiences, which may have influenced their engagement with or perception of the intervention. Also, the analyses of the subgroups based on demographics were conducted but are not reported here due to space constraints and will be explored in future work. Future research should also assess the intervention in pairs (birthing person and their chosen companion) to determine the birth companions’ perceptions of the feasibility and acceptability. In addition, because of the post-test-only design of the study (to avoid fatigue), we did not have any pre-test scores for cognitive variables (self-efficacy and agency). This means that there may have been significant differences in these scores before and after exposure. However, even without this additional data, the results suggests that there is a need for more content and specific messages that would influence change in these areas for BWBP. This pilot study needs expansion with more scenarios that reflect the myriad of interpersonal communication challenges faced by BWBP during delivery hospitalization.

## 5. Conclusions

Despite the social and cultural demographics of the United States becoming increasingly diverse, institutional childbirth education has not changed in over 20 years [37,38]. This study proposes a novel multimedia learning intervention that Black birthing people and their birth companions can use as a catalyst for change to improve Black maternal health disparities. We conducted a mixed-method evaluation of the intervention, and the results suggest that these multimedia assets would be feasible, acceptable, and appropriate for BWBP and their birth companions.

## Figures and Tables

**Table 1:** Demographic characteristics of participants.

Individual-Level Variable	n	%	M	SD
Age	135		29.8	6.8
18–24	34	25.2		
25–34	65	48.1		
35–44	36	26.7		
Residing State (Top 5)	135			
Texas	21	15.6		
Louisiana	12	8.9		
Georgia	9	6.7		
North Carolina	8	5.9		
Michigan	8	5.9		
Level of Education	135			
Less than high school	2	1.5		
Some high school	5	3.7		
High school (e.g., GED)	34	25.2		
Some college, but no degree	35	25.9		
Associate degree	23	17.0		
College (e.g., B.A. or B.S.)	20	14.8		
Some graduate school, but no degree	1	0.7		
Graduate school (e.g., M.S., M.D., or Ph.D.)	15	11.1		
Annual Household Income	135			
Less than USD 20,000	27	20.0		
USD 20,000–USD 44,999	42	31.1		
USD 45,000–USD 139,999	49	36.3		
USD 140,000–USD 149,999	3	2.2		
USD 150,000–USD 199,999	7	5.2		
USD 102,001–USD 105,800	2	1.5		
USD 200,000+	4	3.0		
No. of Children	135		2.1	1.1
One	47	34.8		
Two	52	38.5		
Three	20	14.8		
Four	14	10.4		
Five	2	1.5		
Marital Status When Giving Birth	135	99.3		
Unmarried with a partner	72	53.3		
Unmarried with no partner	9	6.7		
Married	50	37.0		
Divorced	2	1.5		

**Table 2:** Measures and results.

	M (Scale Range)	SD
Feasibility of Intervention Measure		
Animation	4.30 (1–5)	0.79
Game	4.30 (1–5)	0.79
Acceptability of Intervention Measure		
Animation	4.10 (1–5)	0.82
Game	4.30 (1–5)	0.70
Intervention Appropriateness Measure		
Animation	4.36 (1–5)	0.66
Game	4.29 (1–5)	0.76
Cognitive and Behavioral outcomes		
Self-efficacy	5.75 (1–7)	1.30
Agency	5.22 (1–7)	1.14
Intention to use animation/game for future births	4.08 (1–5)	1.24
Intention to share information	3.46 (1–4)	0.57

Note: M = mean, SD = standard deviation.

**Table 3:** Pre-test and post-test knowledge scores.

Item	Pre-Test M (SD)	Post-Test M (SD)	*p*-Value (One-Sided)
1. Black women are 3–5 times more likely to die from pregnancy-related trauma than White women.	0.66 (0.74)	0.84 (0.37)	<0.001
2. Social support can improve birth outcomes for birthing people and their babies.	0.82 (0.39)	0.89 (0.32)	0.04
3. Inadequate communication can make women more at risk for poorer health outcomes.	0.73 (0.45)	0.88 (0.33)	<0.001
4. Listening to the birth person’s needs and advocating for them to medical providers, if necessary, is an important skill for a birth companion or support person.	0.87 (0.33)	0.92 (0.28)	0.07

Note: M = mean, SD = standard deviation.

## Data Availability

Data are available upon reasonable request from the corresponding author.

## Abbreviations

The following abbreviations are used in this manuscript:

BWBP	Black Women and Birthing People
